# The Novel miRNA N-72 Regulates EGF-Induced Migration of Human Amnion Mesenchymal Stem Cells by Targeting MMP2

**DOI:** 10.3390/ijms19051363

**Published:** 2018-05-04

**Authors:** Ying Li, Dianbao Zhang, Meng Chen, Rui Wang, Tao Zhang, Feng Zhao, Xuewen Lin, Xining Pang

**Affiliations:** Department of Stem Cells and Regenerative Medicine, Key Laboratory of Cell Biology, National Health Commission of China, and Key Laboratory of Medical Cell Biology, Ministry of Education of China, China Medical University, Shenyang 110122, China; liying@cmu.edu.cn (Y.L.); zhangdianbao@gmail.com (D.Z.); chenmeng@cmu.edu.cn (M.C.); rwang@cmu.edu.cn (R.W.); zhangt@cmu.edu.cn (T.Z.); fzhao@cmu.edu.cn (F.Z.); xwlin@cmu.edu.cn (X.L.)

**Keywords:** novel microRNA, N-72, EGF, cell migration, hAMSC, MMP2

## Abstract

Human amnion mesenchymal stem cells (hAMSCs) are promising sources of stem cells in regenerative medicine. The migration stimulated by cytokines is critical for mesenchymal stem cells (MSCs)-based cytotherapy, while the regulatory mechanisms of EGF (epidermal growth factor)-induced hAMSC migration are largely unclear. Here, a novel miRNA N-72 (GenBank accession number: MH269369) has been discovered, and its function on EGF-induced migration in hAMSCs was investigated. High-purity hAMSCs were isolated and cultured in vitro, which were characterized by flow cytometry and trilineage differentiation. The N-72 located on chromosome three was conserved, and pri-N-72 owned the ability to form a stem-loop secondary structure, which was predicated by bioinformatic programs. The expression of mature N-72 was verified in several human cells including hAMSC by real-time PCR. In EGF-stimulated hAMSC, N-72 showed a significant reduction in a PI3K and p38 MAPK-dependent manner, and N-72 mimics transfection-inhibited EGF-induced migration, which was verified by scratch assay and transwell assay. Further, the predicated target gene MMP2 was proved to be a direct target of N-72 via luciferase reporter assay, real-time PCR, and Western blotting. The results that MMP2 silencing repressed hAMSC migration suggested MMP2 as a functional downstream target of N-72. In summary, we have discovered the novel N-72, and it was crucial for EGF-induced migration by targeting MMP2 in hAMSCs.

## 1. Introduction

Cytotherapy using mesenchymal stem cells (MSCs) provides novel strategies for the treatment of various diseases, and MSCs have been isolated and expanded in vitro from different tissues such as bone marrow, adipose tissue, and amnion tissue [1,2,3]. Usually, MSC are administered by intravenous or arterial infusion, intraperitoneal injection, and placing cells near lesions [4,5,6]. Therefore, the migration of MSCs to diseased regions is critical for their clinical application. This migration could be stimulated by the cytokines and chemokines secreted from diseased regions such as TGF-β, VEGF, and EGF (epidermal growth factor) [4,7]. EGF could enhance therapeutic potentials including the motility of MSCs, and recent studies demonstrated that human amnion mesenchymal stem cells (hAMSCs) show great differentiation and proliferation potential, as well as other remarkable features that could serve as an outstanding alternative source of stem cells in regenerative medicine [8], while the regulatory mechanisms of EGF-induced hAMSC migration are unclear.

miRNAs are endogenous non-coding small RNAs (18–25 nucleotides long) that regulate gene expression at transcriptional and translational levels, playing important roles in different biological processes, including cell migration [9,10,11]. Recently, several miRNAs have been proved to be critical in MSC migration. miR-375 inhibited the migration of HGF-elicited rat bone marrow-derived MSC by downregulating Akt signaling [4], miR-211 enhanced migration in human and rat bone marrow-derived MSC [12], and miR-335 orchestrated cell migration of MSCs from human bone marrow, adipose tissue, and articular cartilage [13]. Nonetheless, the mechanisms of miRNA regulation in hAMSC migration are largely unknown. In our previous studies, non-coding RNA profiling by high throughput sequencing (GEO accession number: GSE65342) revealed various novel miRNA candidates, providing us more opportunities to access the regulatory mechanisms of hAMSC migration.

In this study, we aimed to identify and characterize the novel miRNA N-72, and investigate the role of N-72 in EGF-induced hAMSC migration, as well as the underlying mechanisms, which might benefit the future therapy using hAMSCs.

## 2. Results

### 2.1. Characterization and Differentiation Capacity of hAMSCs

**Figure 1:**
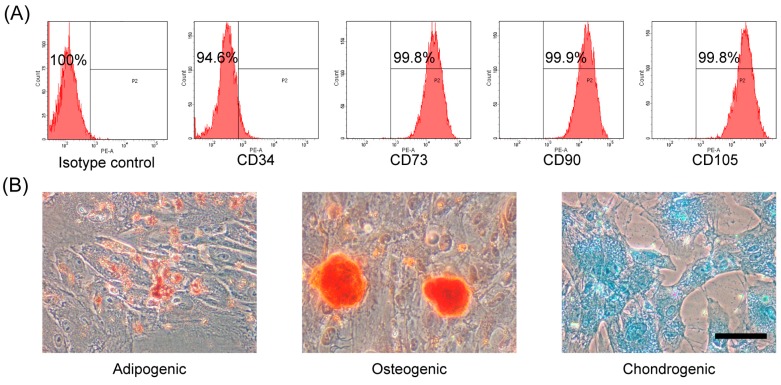
Characterization and differentiation capacity of human amnion mesenchymal stem cells (hAMSCs). (**A**) The hAMSC was characterized by cell surface markers CD34, CD73, CD90, and CD105 using flow cytometry; (**B**) The representative images of adipogenic, osteogenic, and chondrogenic differentiation of hAMSC in vitro. Scale bar indicates 50 μm.

To identify hAMSCs and determine their cell purity, the isolated primary hAMSCs were cultured to passage three, and flow cytometry was applied to analyze cell surface markers. On the basis of the International Society for Cellular Therapy position statement, MSCs should be positive for CD73, CD90, and CD105, and negative for CD34 [14]. As shown in Figure 1A, hAMSCs were above 99% positive for CD73, CD90, and CD105, and below 6% positive for CD34. To investigate the feasibility of inducing hAMSCs to trilineage differentiation in vitro, growth factor-based standard induction and stain methods were carried out. As shown by the representative images in Figure 1B, after induction, most of the derived cells changed phenotype toward a differentiated cell. Further, the adipogenic differentiation cells stored lipid drops inside the cytoplasm (stained with Oil Red O), the osteogenic differentiation cells accumulated calcium (stained with Alizarin Red S), and the chondrogenic differentiation cells produced specific proteoglycan (stained with Alcian Blue). The cells subjected to the non-induced protocol were evidenced by negative staining as negative control. These results suggested that high-purity hAMSCs had been obtained, which was consistent with our previous studies [2].

### 2.2. Discovery of the Novel miRNA N-72

**Figure 2:**
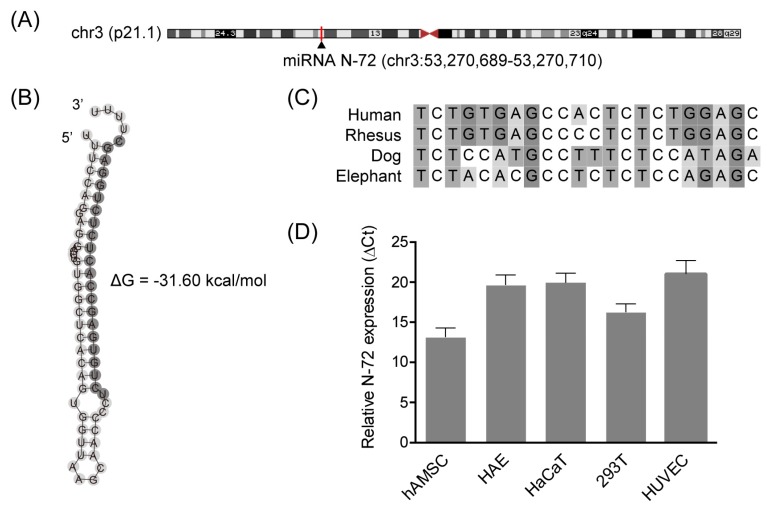
Discovery of the novel miRNA N-72. (**A**) The chromosome location of N-72; (**B**) The predicted secondary structure of pri-N-72, the mature sequence was in gray; (**C**) The conservation analysis of N-72; (**D**) The expression of N-72 in different types of cells including hAMSC, HAE, HaCaT, 293T, and HUVEC, relative to U6.

From the candidate miRNAs revealed by our previous non-coding RNA profiling (GEO accession number: GSE65342), we identified N-72 (5′-TCTGTGAGCCACTCTCTGGAGC-3′, GenBank accession number: MH269369), which was encoded by a gene located on an intergenic region, chr3:53270689-53270710 (Figure 2A). The secondary structure of pri-miRNA is very important for the maturity of miRNA; the potential stem-loop second structure of pri-N-72 showed low folding energy, which was predicted using the RNAfold program (Figure 2B). The N-72 sequence was analyzed using NCBI BLAST and ClustalW among different species, the results showed that N-72 is conserved in several mammals, such as rhesus monkey, dog, and elephant (Figure 2C). In hAMSC, the expression of N-72 was confirmed by real-time PCR with specific stem-loop primers (Figure 2D). Further, N-72 was also proved to be expressed in various human cell types, including amnion epithelial cells (HAE), skin keratinocytes (HaCaT), embryonic kidney cells (293T), and umbilical vein endothelial cells (HUVEC), as shown in Figure 2D. These data provided sufficient evidence for the discovery of the novel miRNA N-72.

### 2.3. N-72 Was Downregulated by EGF Treatment

**Figure 3:**
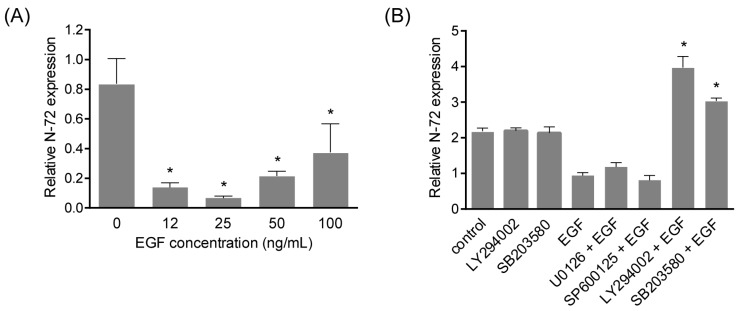
N-72 was downregulated by epidermal growth factor (EGF) treatment. (**A**) The expression of N-72 in hAMSC treated with EGF at different concentrations, relative to the 0 group; (**B**) The effect of different pathway inhibitors (U0126 for MEK1/2, SP600125 for JNK, LY294002 for PI3K, and SB203580 for p38 MAPK) on the expression of N-72, relative to the EGF group. * *p* < 0.05 compared with the 0 (**A**) or control (**B**) groups.

To investigate N-72 expression changes during EGF-induced migration, quantitative real-time PCR was carried out to detect N-72 in hAMSCs treated with EGF at different concentrations. According to the results, N-72 expression displayed a concentration-dependent manner, it was reduced to 0.14-fold at 24 h after EGF treatment at 12 ng/mL, while the repression effect was weakened when EGF concentration reached 50 ng/mL and 100 ng/mL (Figure 3A). To further confirm the regulation of N-72 by EGF, the inhibitory effect of EGF on N-72 was examined after pretreatment with EGF signaling inhibitors (U0126 for MEK1/2, SP600125 for JNK, LY294002 for PI3K, SB203580 for p38 MAPK) [15]. As shown in Figure 3B, EGF-induced N-72 reduction was significantly reversed by LY294002 and SB203580 treatment, indicating that N-72 was suppressed by EGF in a PI3K and p38 MAPK-dependent manner in hAMSCs.

### 2.4. N-72 Inhibited EGF-Induced Cell Migration

**Figure 4:**
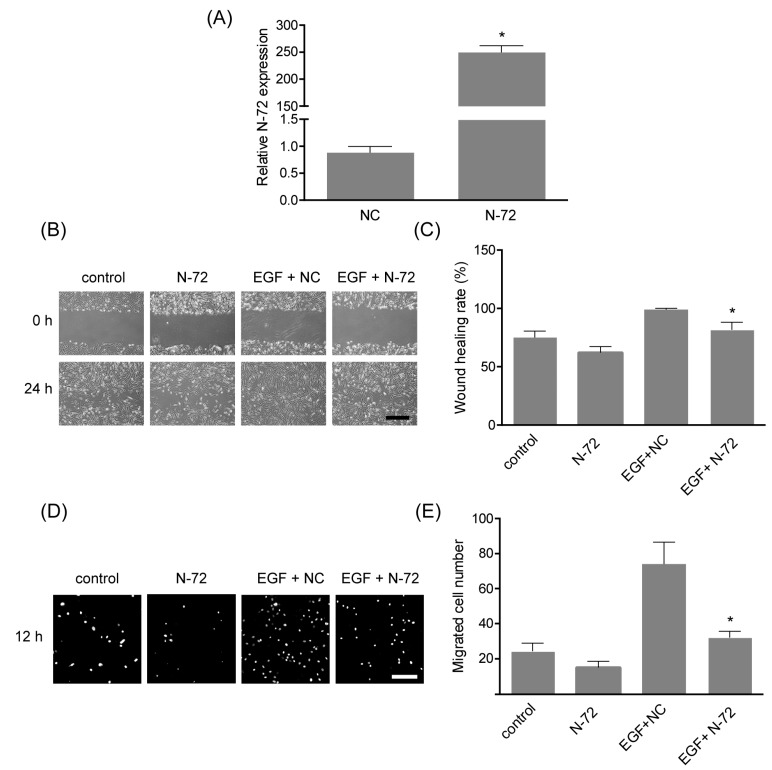
N-72 inhibited EGF-induced hAMSC migration. (**A**) The transfection efficiency of N-72 mimics in hAMSCs was detected by real-time PCR at 48 h after transfection, relative to the negative control (NC) group; (**B**) The cell migration was evaluated by in vitro scratch assay over 12 h; (**C**) The quantification of scratch assay, the wound-healing rate was determined by healing area/wound area × 100%; (**D**) Transwell assay was carried out to detect cell migration over 12 h; (**E**) The quantification of transwell assay, cell migration, was expressed by transmigrated cell number. * *p* < 0.05 compared with the NC (**A**) or EGF+NC (**C**,**E**) groups. Scale bar indicates 100 μm.

To study the role of N-72 in EGF-induced migration, hAMSCs were transfected with N-72 mimics or scramble NC (negative control) and subsequently stimulated with 10 ng/mL EGF for 12 h. The forced expression of N-72 was verified by real-time PCR (Figure 4A). The results of scratch assay presented the enhanced migration of hAMSCs treated with EGF for 12 h, compared with the control group, while it was significantly attenuated by N-72 overexpression (Figure 4B,C). Further, the data obtained from transwell assay revealed that the forced expression of N-72 significantly inhibited the EGF-induced migration of hAMSCs, which was consistent with the results of a scratch assay (Figure 4D,E). In brief, these findings suggested that N-72 might act as a key modulator of EGF-induced migration in hAMSCs.

### 2.5. MMP2 Was a Direct Target of N-72

**Figure 5:**
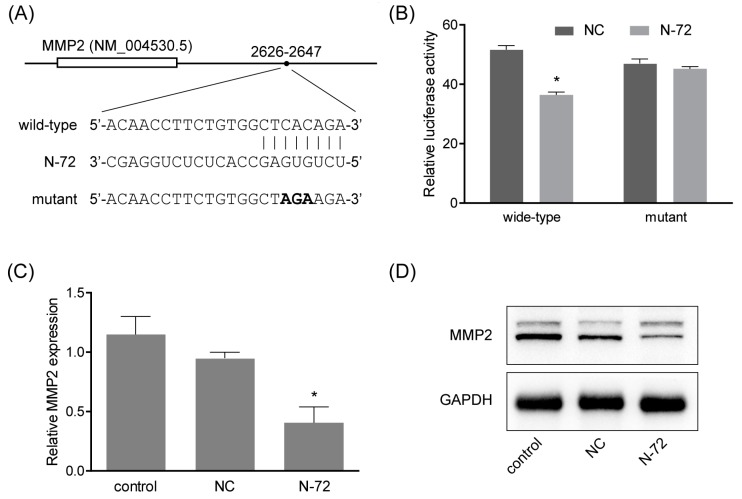
MMP2 is a direct target of N-72. (**A**) The potential N-72 binding site in MMP2 3′-UTR (wild-type) and the mutation (mutant in bold) for dual-luciferase reporter assay; (**B**) The interaction of N-72 and the potential binding site was assessed by dual-luciferase reporter assay; (**C**) The mRNA level of MMP2 in hAMSC transfected with N-72 mimics was analyzed by real-time PCR, relative to the NC group; (**D**) The protein level of MMP2 in hAMSC transfected with N-72 mimics was analyzed by Western blotting. * *p* < 0.05 compared with the NC group.

To gain more insight into the underlying mechanisms by which N-72 exerts its function, the miRanda program was applied on the prediction of target genes that harbored putative binding sites of N-72 within 3′-UTR of their mRNA. As shown in Figure 5A, MMP2, a member of matrix metalloproteinase involved in the breakdown of the extracellular matrix and cytoskeleton rearrangement, was sought out as a potential target gene of N-72, with a putative target sequence at position 2626–2647 (GenBank accession number: NM_004530.5). To further explore whether N-72 directly targets MMP2, a dual luciferase reporter assay was applied. The luciferase reporters containing either a wild-type or mutant binding site were constructed (Figure 5A) and co-transfected with N-72 mimics or NC into 293T cells, and the results showed that, compared with NC, N-72 remarkably decreased the luciferase activity of the wide-type construct, while no significant change of luciferase activity was detected in the mutant reporter (Figure 5B). Notably, the mRNA level of MMP2 could be suppressed by N-72 in hAMSCs, which was confirmed in the protein level by Western blotting (Figure 5C,D). In summary, these data suggested that MMP2 was an authentic target of N-72, which could negatively regulate MMP2 in a post-transcriptional manner in hAMSCs.

### 2.6. MMP2 Was Involved in EGF-Induced Cell Migration

**Figure 6:**
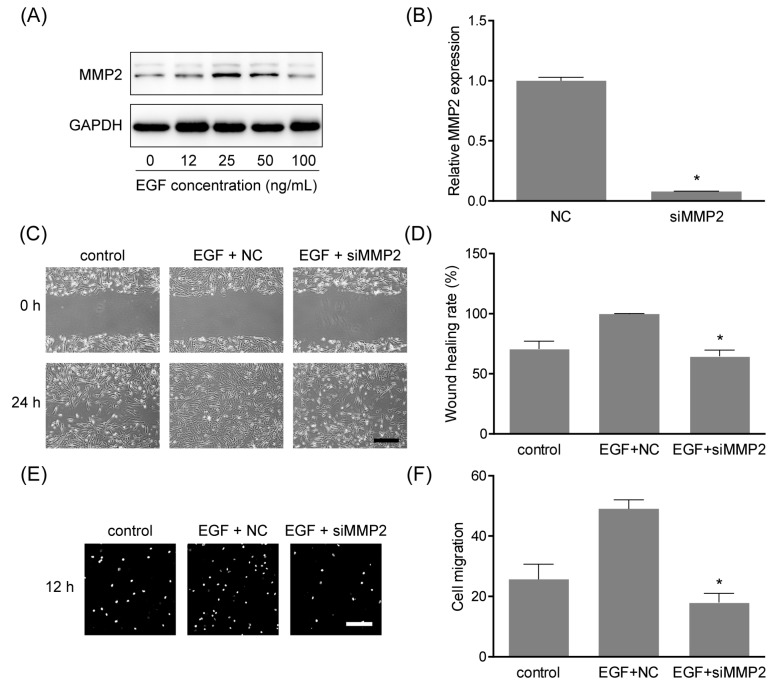
MMP2 was involved in EGF-induced cell migration. (**A**) MMP2 was elevated by EGF treatment in hAMSCs; (**B**) MMP2 silencing efficiency using siRNA was detected by real-time PCR, relative to the NC group; (**C**) The cell migration was evaluated by in vitro scratch assay over 12 h; (**D**) The quantification of scratch assay, the wound-healing rate, was determined by healing the area/wound area × 100%; (**E**) Transwell assay was carried out to detect cell migration over 12 h; (**F**) The quantification of transwell assay, cell migration was expressed by transmigrated cell number. * *p* < 0.05 compared with the NC (**B**) or EGF+NC (**D**,**F**) groups. Scale bar indicates 100 μm.

Previous studies demonstrated that MMP2 could facilitate the migration of MSCs [16,17], while it is unclear in hAMSCs. Thus, the expression of MMP2 in hAMSCs treated with EGF at different concentrations was examined by Western blotting. As shown in Figure 6A, MMP2 was elevated by stimulation of EGF, in a similar concentration-dependent manner as N-72 (Figure 3A). To explore the function of MMP2 in hAMSC migration, MMP2 was knocked down using siRNA silencing (Figure 6B). The results of scratch assay in Figure 6C,D revealed that MMP2 silencing significantly reduced the cell migration of hAMSC, and it was further verified by transwell assay (Figure 6E,F). These results clarified the important role of MMP2 in hAMSC migration, which was a functional downstream target of N-72.

## 3. Discussion

In the present study, we identified and characterized a novel miRNA, N-72, in hAMSCs and other types of cells. The N-72 precursor exhibited a stem-loop secondary folding structure with low folding energy, which is essential for the miRNA biogenesis [18]. The sequence alignment revealed that N-72 is transcribed and derived from an intergenic region between TKT and DCP1A located on chromosome 3, and the mature sequence of N-72 was conserved in several mammals. Further, the expression of N-72 was verified in several different human cells, including hAMSCs.

Several researchers have suggested that miRNA plays key roles in MSC migration. In rat and human MSCs derived from bone marrow, miR-211 overexpression could rescue the aging-impaired migration, and thus increase retention and enhance therapeutic effects in a rat myocardial infarction model, via targeting STAT5A [12]. The expression of miR-335 was high in undifferentiated multipotent hMSCs compared with their differentiated cell progeny, and it was proved to play a central role in the gene regulatory network that controls the cellular movement, partly through the target RUNX2 [13]. During HGF-elicited migration of MSC, miR-375 was downregulated, and its overexpression decreased cell migration through repressing PDK1 to reduce Akt signaling [4]. Here, we also found the remarkable reduction of N-72 in hAMSC stimulated with EGF, in a PI3K and p38 MAPK signaling-dependent manner. Functionally, N-72 overexpression significantly inhibited EGF-induced cell movement of hAMSC in scratch assay and transwell assay; in other words, N-72 treatment could bypass EGF-induced hAMSC migration. These results indicated that N-72 might be crucial for hAMSC migration and represent a potential modification strategy for cytotherapy using hAMSC.

Generally, miRNA plays important roles in regulating targeted genes at the post-transcriptional level, through directly targeting their 3′-UTR to induce mRNA degradation or suppress mRNA translation [19]. Using the miRanda algorithm, MMP2 was sought out as a potential target gene with an 8-mer binding site in its 3′-UTR. Subsequently, the interaction of N-72 with MMP2 mRNA was detected by luciferase reporter assay, and confirmed by real-time PCR and Western blotting. The expression of MMP2 was inhibited by N-72 mimics at both mRNA and protein levels, indicating that N-72 repressed MMP2 partly through mRNA degradation. In previous studies, MMP2 was demonstrated to be crucial to the maintenance of migration in MSCs [16,20]. Here, MMP2 expression was proved to be upregulated in a concentration-dependent manner under EGF stimulation, and MMP2 silencing significantly reduced the migration capacity of hAMSCs. These data revealed that MMP2 was a functional downstream target gene of N-72, playing key regulatory roles in EGF-induced migration in hAMSCs.

In summary, we identified and characterized the novel N-72 in hAMSCs, which played important roles during EGF-induced migration by targeting MMP2. Further studies are needed to determine whether N-72 could be a modification target to improve cytotherapy.

## 4. Materials and Methods

### 4.1. hAMSC Isolation and Cell Culture

Human amnion of normal pregnancies was obtained after caesarean section with informed consent as approved by the Ethical Committee of China Medical University (9 January 2014). The isolation of hAMSCs was performed as described [2], using the enzymatic treatment of amnion with collagenase IV (Sigma, Saint Louis, MO, USA) and DNase I (Takara, Dalian, China) after manual separation from chorion. hAMSCs were cultured in DMEM/F12 (Hyclone, Logan, UT, USA) supplemented with 10% FBS (Hyclone) and 1% Penicillin streptomycin (Gibco, Carlsbad, CA, USA). HAE was isolated as described and culture in the same medium as the hAMSCs [21]. HaCaT, 293T, and HUVEC were provided by Chundi He, Ziwei Miao and Hongmei Yang separately, and cultured in DMEM (Hyclone) containing 10% FBS and 1% Penicillin streptomycin. For the treatments with different pathway inhibitors (U0126 for MEK1/2, SP600125 for JNK, LY294002 for PI3K, SB203580 for p38 MAPK), the cells were pretreated with the inhibitors at 1 h before EGF stimulation.

### 4.2. Flow Cytometry Analysis

The immunophenotype of the culture-expanded hAMSCs was analyzed by flow cytometry. The cell suspensions were incubated for 30 min at room temperature with PE-conjugated antibodies against human antigens CD14, CD34, CD45, CD90, and CD105, and PE-conjugated non-specific mouse IgG was used as isotype control. The samples were analyzed by a flow cytometer LSRFortessa (BD Biosciences, Franklin Lakes, NJ, USA). All of the antibodies were purchased from BioLegend (San Diego, CA, USA).

### 4.3. Trilineage Differentiation in Vitro

The adipogenic, osteogenic, and chondrogenic differentiation capacity of hAMSC was determined using StemPro Adipogenesis/Osteogenesis/Chondrogenesis Differentiation Kits (Gibco) according to the manufacturer’s instructions for 16 days, 22 days, and 16 days separately. Adipogenic differentiation was visualized after staining with Oil Red O (Cyagen, Suzhou, China), osteogenesis differentiation was stained with Alizarin Red S (Cyagen), and chondrogenesis differentiation was stained with Alcian Blue solution (Sigma-Aldrich, Shanghai, China).

### 4.4. Bioinformatic Analysis

Based on the results of our previous next-generation sequencing (GEO accession: GSE 65342), the chromosome location of N-72 was analyzed by NCBI BLAST and UCSC Genome Browser; the conservation of N-72 was analyzed using NCBI BLAST and ClustalW in MEGA 7 [22]; the potential for putative small RNA to form a stem-loop was examined using the RNAfold webserver (http://rna.tbi.univie.ac.at) [23]. The targets of N-72 were predicted using miRanda [24].

### 4.5. Quantitative Real-Time PCR

Total RNA was isolated using RNAiso (Takara) and quantified by NanoDrop 2000C spectrophotometer (Thermo, Wilmington, DE, USA). For miRNA detection, reverse transcription PCR was performed using specific stem-loop primers, M-MLV Reverse Transcriptase, and Recombinant RNasin Ribonuclease Inhibitor (Promega, Madison, WI, USA); for mRNA, cDNA was synthesized using a PrimeScript RT reagent Kit with gDNA Eraser (Takara). Quantitative real-time PCR was applied using SYBR Premix Ex Taq II (Takara) in an ABI 7500 Real-Time PCR System. Fold changes were calculated using the ΔCt method (N-72 in different types of cells) and ΔΔCt method (others). The primer sequences were as follows: 5′-GTCGTATCCAGTGCAGGGTCCGAGGTATTCGCACTGGATACGACGCTCCA-3′ for N-72 reverse transcription; 5′-CGAATTTGCGTGTCATCCT-3′ for U6 reverse transcription; 5′-GCGTCTGTGAGCCACTCTC-3′ (forward) and 5′-GTGCAGGGTCCGAGGT-3′ (reverse) for N-72; 5′-CTCGCTTCGGCAGCACATA-3′ (forward) and 5′-CGAATTTGCGTGTCATCCT-3′ (reverse) for U6 as an internal control for miRNA; 5′-CCCACTGCGGTTTTCTCGAAT-3′ (forward) and 5′-CAAAGGGGTATCCATCGCCAT-3′ (reverse) for human MMP2; 5′-GCACCGTCAAGGCTGAGAAC-3′ (forward) and 5′-TGGTGAAGACGCCAGTGGA-3′ (reverse) for human GAPDH as an internal control for mRNA.

### 4.6. miRNA and siRNA Transfection

miRNA and siRNA transfection was carried out using a Lipofectmine RNAiMAX Transfection Reagent (Invitrogen, Shanghai, China) as described [25]. Oligonucleotides were synthesized by GenePharma (Shagnhai, China) and the sequences were as follows: 5′-UCUGUGAGCCACUCUCUGGAGC-3′ for N-72 mimics, 5′-GUGGCCAACUACAACUUCUTT-3′ for MMP2 siRNA, and 5′-UUCUCCGAACGUGUCACGUTT-3′ for NC.

### 4.7. Transwell Migration Assay

The migration ability of hAMSCs was assessed using transwell. The cells were seeded on eight-μm Transwells (6.5 mm diameter, Corning, Tewksbury, MA, USA) at 6 × 10^4^ cells per well in serum-free medium, and the complete medium was added to the lower chamber. Then, 24 h after incubation, the upper surface of the filter was gently wiped with a cotton swab. The cells that migrated through the filter were stained with DAPI and visualized by an Observer A1 microscope (Zeiss, Oberkochen, Germany). The graphs were analyzed using ImagePro Plus 6.0 software (Media Cybernetics, Rockville, MD, USA).

### 4.8. In Vitro Scratch Assay

The cells were cultured to confluence in six-well plates, and the cell monolayers were scratched using a sterile 200-μL pipette tip. Images were taken at 0 h and 12 h after scratch using an Olympus CKX31 microscope (Tokyo, Japan), and analyzed using TScratch software (CSElab, Zurich, Switzerland). The wound-healing rate was determined by healing area/wound area × 100%.

### 4.9. Dual-luciferase Reporter Assay

The 647 bp of MMP2 3′-UTR containing the putative binding site for N-72 was cloned into pmirGLO Dual-Luciferase miRNA Target Expression Vector (Promega) using forward primer 5′-CgagctcCTCCACTGCCTTCGATACACC-3′ with a SacI site (lowercase) and reverse primer 5′-GCtctagaGAGACTCGGTAGGGACATGC-3′ with an XbaI site (lowercase). The mutation in the putative seed region was achieved using a Site-directed Gene Mutagenesis Kit (Beyotime, Haimen, China) and the following primers: 5′-CTTTCACAACCTTCTGTGGCTAGAAGAACCCTTGGAGCCAATGG-3′ (forward) and 5′-CCATTGGCTCCAAGGGTTCTTCTAGCCACAGAAGGTTGTGAAAG-3′ (reverse). 293T cells seeded on 24-well plates in DMEM (Hyclone) containing 10% FBS were co-transfected with either 50 nmol of N-72 mimics or NC and 200 ng of endo-free purified (Endo-Free Plasmid Midi Kit, Omega bio-tec, Guangzhou, China) wide-type or mutant plasmid using Lipofectamine 3000 Transfection Reagent (Invitrogen). The relative luciferase activities were determined at 48 h after transfection using the Dual Glo Luciferase Assay System (Promega) on GloMax Multi Plus Detection System (Promega).

### 4.10. Western Blotting

Total protein lysates were extracted using RIPA lysis buffer (Beyotime) supplemented with protease inhibitors (Roche, Penzberg, Germany), and the concentrations of the samples were determined using a BCA Protein Assay Kit (Beyotime). Equal amounts of total proteins were separated by SDS-PAGE (Transgen, Beijing, China), transferred to the PVDF membrane (Millipore, Billerica, MA, USA) and probed with a rabbit anti-MMP2 polyclonal antibody (1:2000, Proteintech, Wuhan, China) and mouse anti-GAPDH antibody (1:10,000, Proteintech). After overnight incubation with HRP-conjugated secondary antibodies (1:10,000, Proteintech), protein bands were visualized using Immobilon Western Chemiluminescent HRP Substrate (Millipore) on a Tanon-5200 chemiluminescence detection system (Tanon, Shanghai, China). The bands were analyzed by using Image J software (NIH, Bethesda, MD, USA).

### 4.11. Statistical Analysis

The results were expressed as the mean ± SD from at least three independent experiments. Statistical analysis was performed using Student’s *t*-test or one-way analysis of variance (ANOVA). *p* < 0.05 was considered statistically significant. All of the statistical analyses were performed using GraphPad Prism 6 software (GraphPad Software, Inc., La Jolla, CA USA).
